# Introgression of the *Aedes aegypti* Red-Eye Genetic Sexing Strains Into Different Genomic Backgrounds for Sterile Insect Technique Applications

**DOI:** 10.3389/fbioe.2022.821428

**Published:** 2022-02-02

**Authors:** Antonios A. Augustinos, Katerina Nikolouli, Lucia Duran de la Fuente, Muhammad Misbah-ul-Haq, Danilo O. Carvalho, Kostas Bourtzis

**Affiliations:** ^1^ Insect Pest Control Laboratory, Joint FAO/IAEA Centre of Nuclear Techniques in Food and Agriculture, Department of Nuclear Sciences and Applications, IAEA Laboratories, Seibersdorf, Austria; ^2^ Nuclear Institute for Food and Agriculture, Peshawar, Pakistan

**Keywords:** area wide integrated pest management, insect pest control, vector control, mosquitoes, yellow fever mosquito

## Abstract

*Aedes aegypti* is an invasive mosquito species and major vector of human arboviruses. A wide variety of control methods have been employed to combat mosquito populations. One of them is the sterile insect technique (SIT) that has recently attracted considerable research efforts due to its proven record of success and the absence of harmful environmental footprints. The efficiency and cost-effectiveness of SIT is significantly enhanced by male-only releases. For mosquito SIT, male-only releases are ideally needed since females bite, blood-feed and transmit the pathogens. *Ae. aegypti* genetic sexing strains (GSS) have recently become available and are based on eye colour mutations that were chosen as selectable markers. These genetic sexing strains were developed through classical genetics and it was shown to be subjected to genetic recombination, a phenomenon that is not suppressed in males as is the case in many Diptera. The genetic stability of these GSS was strengthened by the induction and isolation of radiation-induced inversions. In this study, we used the red eye mutation and the inversion Inv35 line of the *Ae. aegypti* red-eye GSS s and introgressed them in six different genomic backgrounds to develop GSS with the respective local genomic backgrounds. Our goal was to assess whether the recombination frequencies in the strains with and without the inversion are affected by the different genomic backgrounds. In all cases the recombination events were suppressed in all Inv35 GSS strains, thus indicating that the genomic background does not negatively affect the inversion result. Absence of any effect that could be ascribed to genetic differences, enables the introgression of the key elements of the GSS into the local genomic background prior to release to the target areas. Maintaining the local background increases the chances for successful matings between released males and wild females and addresses potential regulatory concerns regarding biosafety and biosecurity.

## Introduction

Arthropod-borne viruses or “arboviruses” transmitted by *Aedes* spp. mosquitoes are accountable for the emergence of human epidemic diseases across the globe (Weaver and Reisen 2010; Lucey and Gostin 2016; Siraj et al., 2017; Wilder-Smith et al., 2017). Zika, dengue, yellow fever, and chikungunya viruses infect humans by the bite of an infected *Aedes aegypti* L. (Diptera: Culicidae) mosquito and result in a diverse array of clinical symptoms and implications ranging from systemic febrile illnesses to neurological or cerebrovascular diseases and death (Bhatt et al., 2013; Beckham and Tyler 2015; Paixão et al., 2018). *Ae. aegypti* has successfully spread in tropic and subtropic zones worldwide (Kraemer et al., 2015). It is daylight-active, thrives in urban and peri-urban areas, feeds exclusively on human blood multiple times during a gonotrophic cycle, and shows high susceptibility to arboviruses (Scott and Takken 2012; Wilder-Smith et al., 2017; Ryan et al., 2019). Its ability to breed in human-made breeding settings facilitates the increase of the vector’s population and fuels the spread of the vector-borne diseases. Urbanization of rural areas, increase of travelling activities, globalization, and climate change accelerate the invasion potential of *Ae. aegypti* and enhance the viral transmission (Wilder-Smith and Gubler 2008; Bhatt et al., 2013; Struchiner et al., 2015; Ryan et al., 2019; Iwamura et al., 2020).

The lack of effective drugs and vaccines against these arboviruses (apart from the yellow fever vaccine) has shifted the spotlight on the vector population control methods (Achee et al., 2015; Lees et al., 2015; Bourtzis et al., 2016; Flores and O’Neill 2018). Current efforts rely on insecticide applications and elimination of breeding sites; however, these methods have been proved both unsustainable and inefficient. The development of insecticide resistance, the rapid expansion of *Ae. aegypti* populations in urban areas and the inadequate control of the cryptic breeding sites led scientists and communities to pursue environmentally-friendly approaches that would control efficiently the vector populations without compromising sustainability (Lima et al., 2011; Achee et al., 2015; Louis et al., 2016; Moyes et al., 2017).

During the recent years, numerous genetically based approaches have been developed aiming either to modify vector populations (i.e., rendering them resistant in pathogen transmission) or to suppress them below the threshold required for disease transmission (Harris et al., 2012; O’Connor et al., 2012; Alphey et al., 2013; Bellini et al., 2013; Carvalho et al., 2015; Mains et al., 2016; Kittayapong et al., 2018; Kyrou et al., 2018; Kandul et al., 2019; Kittayapong et al., 2019; Zheng et al., 2019; Crawford et al., 2020). Some of the population suppression approaches, including the sterile insect technique (SIT), have been tested in the field with encouraging results (O’Connor et al., 2012; Bourtzis et al., 2014; Bourtzis et al., 2016; Kittayapong et al., 2018; Kandul et al., 2019; Kittayapong et al., 2019; Zheng et al., 2019; Crawford et al., 2020). The SIT which relies on the mass production and release of sterile males, has historically been applied for the control and eradication of insect pest populations (Bushland et al., 1955; Knipling 1955; Klassen et al., 2021). When considering the SIT as part of a mosquito control project, one of the greatest challenges to be addressed is the sex separation and elimination of females (Papathanos et al., 2009; Gilles et al., 2014). Unlike agricultural pests where either bisexual release is the only feasible approach or the accidental release of few females is not considered a major concern, in mosquitoes, release of both males and females is a no go, since adult females create biting nuisance, and are potential disease vectors. Thus, an adequate and robust sex separation system that will reliably separate male and female mosquitoes at a large scale is of critical importance for the implementation of a SIT program (Gilles et al., 2014; Papathanos et al., 2018). In *Ae. aegypti*, sex separation is currently based on the inherent characteristics of the species, i.e., the size dimorphism between male and female pupae and male-specific body parts of adults including genitalia and antennae (Focks 1980; Gunathilaka et al., 2019; Crawford et al., 2020). This approach is rearing-dependent, prone to errors, labor-intensive and appropriate for small-scale operations. Although novel and (semi)-automated methods have been developed, the critical need of a genetic sexing strain (GSS) for *Ae. aegypti* rises as the ideal sex separation method particularly if males and females could potentially be separated at early developmental stages (Gilles et al., 2014; Papathanos et al., 2018). Developing a GSS using classical genetics typically requires a selectable marker (visually detectable or conditionally lethal) and the linkage of the wild type allele of this marker to the Y chromosome or to the sex-determining genetic locus (Franz et al., 2021).

*Aedes* species have homomorphic sex chromosomes and their maleness is defined by a dominant male-determining locus (M locus) of chromosome 1 (Craig and Hickey 1967; Newton et al., 1974; Hall et al., 2015; Aryan et al., 2020; Liu et al., 2020). *Ae. aegypti* males are heterogametic (Mm) while the females are homogametic (mm) for the M-locus (Timoshevskiy et al., 2013). The competence of the selectable marker will in turn determine the robustness of the GSS and in *Ae. aegypti* the ideal marker would reside on chromosome 1, closely linked to the M-locus. In such a strain, male mosquitoes would be heterozygotes and express the “wild-type” phenotype while females would be homozygous for the recessive alleles of the selectable marker expressing the mutated phenotype (Franz et al., 2021). Promising markers that could be used for *Ae. aegypti* GSS development are related to eye colour (Red-eye (*re*) and White-eye (*w*) markers) which are located on chromosome 1 linked to the M-locus and they are fully penetrant and expressive (Bhalla and Craig 1970; Munstermann and Craig 1979). Both markers have been used by our group for the construction of two *Ae. aegypti* GSS, in which males have black eyes and females have either red or white colour eyes (Koskinioti et al., 2020). The *re* and *w* mutant lines were crossed with the wild-type “BRA” strain collected from Brazil and the Red-eye GSS and White-eye GSS were developed. Quality control of both GSSs evidenced no significant differences regarding sex ratio and immature development duration of both sexes. The Red-eye GSS showed outstanding productivity compared to the White-eye GSS and significantly elevated lifespan and flight ability compared to the wild type “BRA” strain (Koskinioti et al., 2020).

The stability of a GSS, in particular under the demanding mass-rearing conditions, is a pivotal factor for its successful implementation in operational population suppression programmes. Instability during mass-rearing conditions is mainly attributed to genetic recombination events. Unlike other Diptera, in *Aedes* mosquitoes, recombination events occur in males almost as frequently as in females, and these events can compromise the GSS stability and lead in breakdown of the GSS due to accumulation of recombinants. Incorporation of recombination-suppressing factors, such as inversions, can improve the stability of a GSS (Franzet al. 2021; Gilles et al., 2014; Zacharopoulou et al., 2017). In *Ae. aegypti* induction of inversions has been shown to suppress recombination between the M locus and morphological markers of chromosome 1 (Bhalla 1973). Using irradiation our group induced inversions in *Ae. aegypti* and showed that irradiation frequency can be suppressed between *re* and the M locus (line 35), while at least two lines in which recombination is suppressed between *w* and the M locus (lines 5 and 35) were identified (Augustinos et al., 2020). Inversion line 35 was incorporated in the Red-eye GSS and White-eye GSS by crossing wild-type males having the recombination suppressor (from the Inv35 line) with females from the two GSSs. Recombination frequencies were measured for consecutive generations under filtered and non-filtered conditions, i.e., removal or not of recombinant progeny from each generation, and recombination was consistently reduced for both strains (Koskinioti et al., 2020).

GSSs may still face issues when released in the field that could lead in performance reduction. The genomic background has been shown to be a driving factor when it comes to mosquito performance. Among others, variation in vector competence, reproductive incompatibility, effects on fitness traits and differences in the reproductive effects of *Wolbachia* infections have been shown to stem from variations in the genomic background of mosquito populations (Bennett et al., 2002; Menge et al., 2005; Axford et al., 2016; Dickson et al., 2016; Campbell et al., 2017; Carvalho et al., 2020; Enkerlin 2021). The success of sterile mosquito releases relies massively on the mating performance of the released males. However local mosquito populations might vary significantly in terms of ecology, biology, and behavior and this could in turn lead to mating barriers which would compromise the efficiency of a SIT programme (Krafsur and Ouma 2021). These barriers can be overcome by developing mosquito GSS that will be integrated into the local genomic background of the release area. In this study the *A. aegypti* red eye mutation and the inversion Inv35, the latter developed previously in the Insect Pest Control Laboratory (IPCL, Seibersdorf, Austria), were introgressed in populations originating from different geographic areas to develop Red-eye GSS and Red-eye GSS/Inv35 strains with local genomic backgrounds and their genetic stability was assessed for several generations (Augustinos et al., 2020; Koskinioti et al., 2020).

## Materials and Methods

### *Ae. aegypti* Strains and Rearing Conditions

The Rexvillle Red Eye strain, which is homozygous for the recessive *re* allele, was used in the present study and was kindly provided by Dr. Margareth Capurro at the Department of Parasitology, University of Sao Paulo, Brazil. In the *re* strain all individuals have red eye color which is evident throughout all developmental stages and it darkens as adults age. Six wild type *Ae. aegypti* strains originated from Brazil (BRA), Indonesia (IDN), Mexico (MEX), Singapore (SGP), Sri Lanka (LKA), and Thailand (THA) were used for the introgression crosses described below and checked for their recombination rates. The *Ae. aegypti* inversion line 35 (Inv35) (Augustinos et al., 2020) was used to incorporate the inversion in all six genomic backgrounds. In all wild-type strains the eye color is dark brown/black and remains stable at all developmental stages. All strains were maintained in the insectary of the Insect Pest Control Laboratory (Joint FAO/IAEA Centre, Seibersdorf, Austria) at 27 ± 1°C, 80% relative humidity and a 12/12 h day/night photoperiod.

Adult mosquitoes were kept in standard (30 × 30 × 30 cm) insect plastic rearing cages (BugDorm-41,515 insect cage) and a 10% sucrose solution was constantly provided. Female mosquitoes were blood-fed with porcine blood twice per week. The blood used was collected in Vienna, Austria during routine slaughtering of pigs in a nationally authorized abattoir, conducted at the highest possible standards strictly following EU laws and regulations. Egg collections were initiated 72 h after the last blood feeding using moistened oviposition papers (white germination paper, Sartorius Stedium Biotech, Austria).

### Crosses

#### Development of Red Eye-GSS Strains in Local Genomic Backgrounds

**FIGURE 1:**
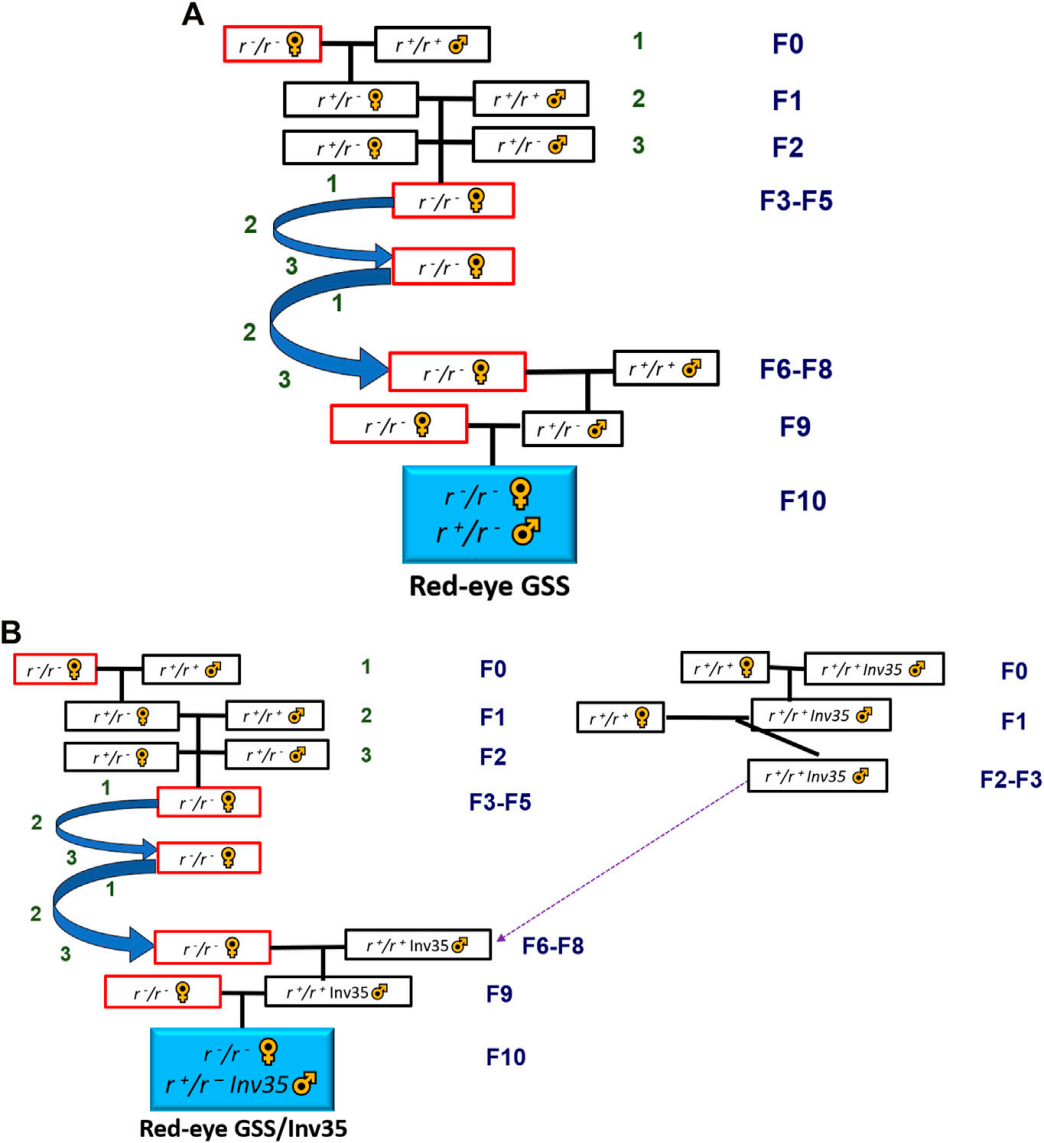
**(A)** Crossing scheme for the introgression of the red eye mutation into a local genomic background, **(B)** Crossing scheme for the introgression of the red eye mutation and the inversion 35 into a local genomic background. Both schemes are simplified and not all genotypes retrieved after each cross are depicted. See Supplementary Methods S1 and S2 for detailed analysis.

Females of the Red Eye strain and males from IDN, SGP and LKA populations were used to initiate the three introgression crosses while the respective crosses with males from BRA, MEX and THA populations are reported in the study of Chen et al. (2021) (Figure 1A).

The introgression of Inv35 in the local genomic backgrounds was initiated independently and it was continued until a semi-introgressed inversion line had been acquired. At that stage, partially Introgressed Inv35 males were crossed with highly introgressed Red Eye females to create a Red-GSS with Inv35 in a local genomic background (Figure 1B). The genomic backgrounds of the introgressed Red Eye females were from BRA, IDN, MEX, SGP, LKA and THA populations. In all cases, fifty females and twenty males were crossed in every generation in a 15 × 15 × 15 cm rearing cage (BugDorm-4M1515). The detailed introgression protocols are provided in the Supplementary Materials S1 and S2.

### Estimation of Recombination Rate

The recombination rate was estimated for all the newly established Red-eye GSS and Red-eye GSS/Inv35. All progeny were screened in every generation and recombinants (males with red eyes and females with black eyes) were recorded and subsequently discarded. At least six generations per strain were screened. Black eye males and red eye females were used to set up the new cages. A minimum number of 1,000 individuals were used to set up the new cages.

### Data Analysis

All statistical analyses were performed using R version 4.0.5 (R Core Team, 2021). The recombination rates between the strains with and without inversion of the same origin and among the different origins represent proportional data and therefore, they were analyzed using a GLM-binomial family and a logit link function (Dunn and Smyth, 2018). In case overdispersion was detected, a Quasi-Binomial model with a logit link function was applied (Demétrio et al., 2014). Analysis of deviance was performed with a Chi-squared test for GLM-Binomial models and with a F-test for GLM-Quasi-Binomial models (Nelder and Wedderburn, 1972). Residuals of the models were checked for normality and homogeneity of variance. Goodness-of-fit of the models was visually inspected with half-normal plots with simulation envelopes (Moral et al., 2017). Emmeans package was used for the pairwise comparisons of the fitted model estimates (Searle et al., 1980).

## Results and Discussion

The *Ae. aegypti* Red-eye GSS has been developed through classical genetics and is based on the *re* morphological marker that has been mapped to chromosome I (Koskinioti et al., 2020). The red eye mutation presents full penetrance and expressivity and the red eye color is evident throughout all developmental stages. Estimation of the recombination frequency between *re* and M locus confirmed that *re* is a recessive, sex-linked gene. Recombination events in *Aedes* species occur both in males and females and, in the case of a GSS under mass-rearing conditions, they can eventually lead in reduced genetic stability and colony collapse (Augustinos et al., 2017; Franz et al., 2021). Elements that suppress recombination between the M locus and the marker are therefore required to be incorporated in the GSS. In the study by Augustinos et al. (2020) an inversion (Inv35) was induced through irradiation aiming to suppress recombination between *re* and the M locus. Indeed, the recombination frequency was significantly suppressed, and the inversion was incorporated in the Red-eye GSS thus creating the Red-eye GSS/Inv35. These two strains were screened for numerous generations and results demonstrated significantly decreased recombination in the Red-eye GSS/Inv35 compared to the original strain (Koskinioti et al., 2020).

Variability in recombination frequencies can be attributed, among other factors, to genomic differences, with chromosomal rearrangements being the most likely reason (Dickson et al., 2016). In the present study, we received six *Ae. aegypti* populations from countries that could be possible target areas of a future operational SIT programme. The red eye mutation line and the inversion Inv35 were introgressed into the six genomic backgrounds, following a crossing scheme that lasted for eleven generations. Our goal was to assess whether the novel genomic background would affect the recombination frequencies that had been estimated in the original GSS. As soon as the introgression crossing scheme was completed and the twelve new strains had been established, the recombination frequencies were evaluated for all strains. A total of 110,799 mosquitoes from 73 generations of 12 strains were screened and recombination frequencies were recorded (Supplementary Materials S3 and S4).

**FIGURE 2:**
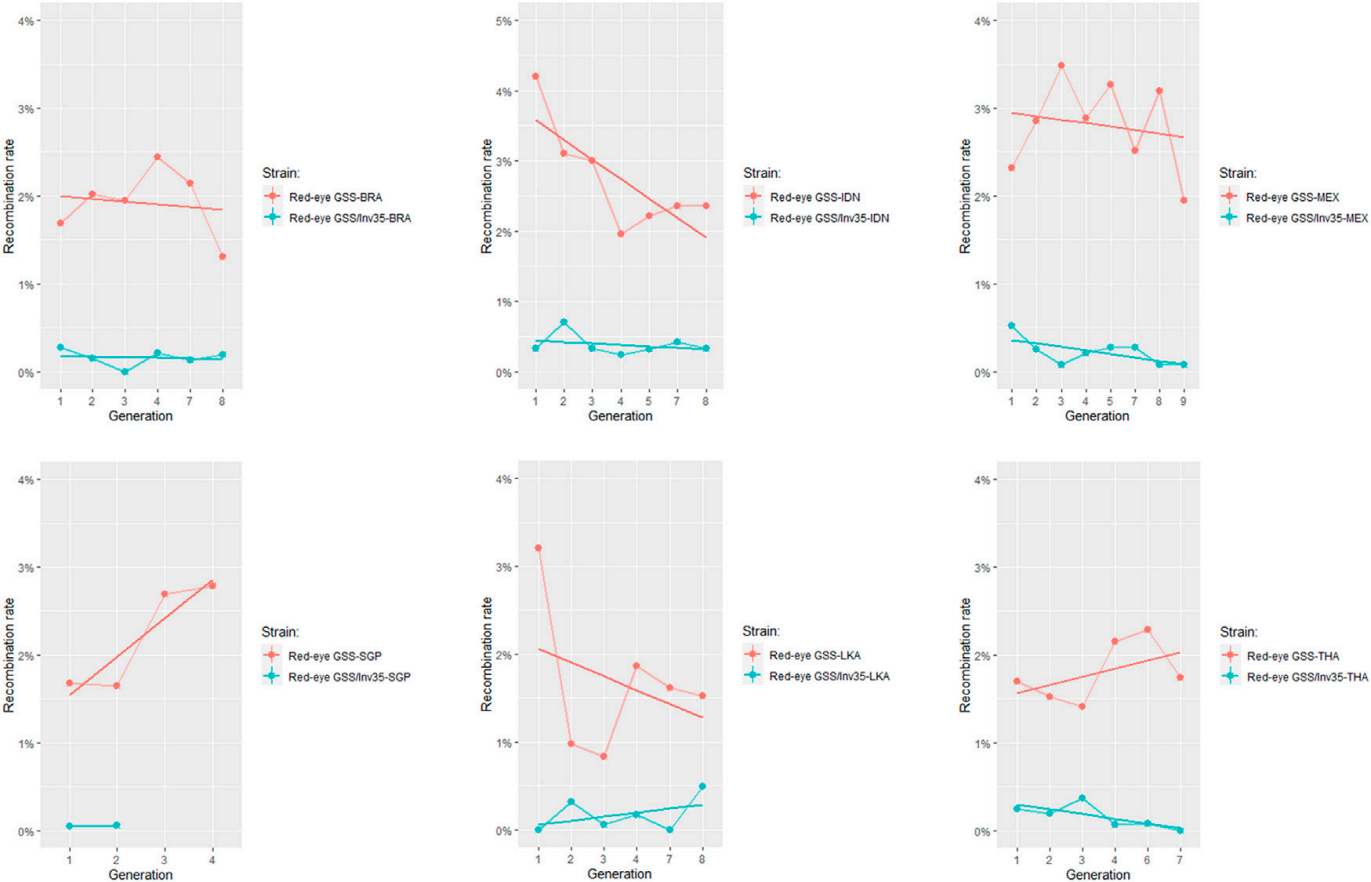
Recombination rates of the Red-eye GSS and Red-eye GSS/Inv35, after their introgression in six local genomic backgrounds. Recombinant males and females were recorded in each generation and results were analyzed with a GLM (binomial family). In all genomic backgrounds, the strain incorporating the inversion had significantly lower recombination rates compared to the respective strains without the inversion. The straight line represents the fitted linear model.

**FIGURE 3:**
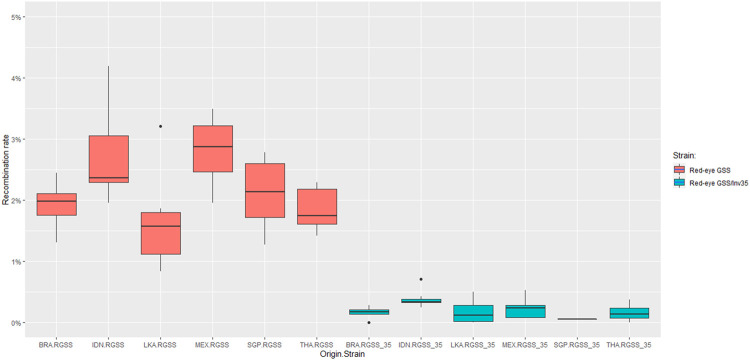
Recombination rates of the Red-eye GSS and Red-eye GSS/Inv35 throughout the course of generations. Generations were used as replicates. No significant effect was detected among the Red-eye GSS/Inv35 strains, indicating that Inv35 suppresses recombination irrespectively of the genomic background.

The recombination frequency was estimated for each genomic background individually and results indicated that the Red-eye GSS/Inv35 presented significantly lower recombination rates compared to the Red-eye GSS throughout the course of generations (Figure 2). Except for Singapore for which data availability is limited, the strains with the inversion were more stable and with significantly lower recombination rates compared to the ones without (Supplementary Material S5). Recombination frequencies were analyzed to check for any possible effect of the genomic background, using data from different generations as replicates. In all genomic backgrounds the recombination rates were significantly lower for the Red-eye GSS/Inv35 (F = 51.375, *df* = 11, *p* < 2.2e-16), thus indicating that the effect of inversion is evident in all genomic backgrounds (Figure 3 and Supplementary Material S6). Assessment of the recombination frequencies among the six Red-Eye GSS/Inv35 strains showed no statistically significant differences, thus suggesting that the inversion suppresses recombination similarly, irrespective of the genomic background (Supplementary Material S6). Interestingly, the pairwise comparisons of the six Red-Eye GSS strains showed an effect of the genomic background on the recombination rates. Red-eye GSS-BRA was shown to be significantly different from the MEX (z = −3.495, *p* = 0.0005) and IDN (z = −3.035, *p* = 0.0024) strains, while the same was also true for the IDN-LKA (z = 3.422, *p* = 0.0006), IDN-THA (z = 2.855, *p* = 0.0043), MEX-LKA (z = −3.889, *p* = 0.0001), and MEX-THA (z = 3.353, *p* = 0.0008) Red-Eye GSS comparisons. However, in some Red-eye GSS strains *per se* the recombination frequencies varied among generations. Results for Indonesia, Mexico, and Sri Lanka showed that there was a statistically significant difference among the tested generations (Supplementary Material S7) which could be attributed to factors such as age, sex and temperature (Augustinos et al., 2020). No variation was detected for the Red-eye GSS from Brazil, Singapore and Thailand. The same conclusion was also reached for the Red-eye GSS/Inv35 strains (Supplementary Material S7). The recombination rates were stable through the course of generations per strain and no statistically significant differences were detected for all the genomic backgrounds which confirmed the robustness and effectiveness of the inversion in suppressing recombination regardless of genomic background and generation. This clearly indicated that the genomic background did not negatively affect the genetic stability of the strains and confirmed the robustness and effectiveness of the inversion in suppressing recombination regardless of genomic background and generation. The Red-eye GSS/Inv35-SGP is the only strain for which availability of data is limited and therefore more generations are required to reach a safe conclusion.

The results of the present study are encouraging, in respect to the genetic stability of Red-eye GSS/Inv35 developed in local genomic backgrounds. However, the biological quality of the newly established GSS needs to be assessed first under laboratory and later in field conditions (Carvalho et al., 2020; Koskinioti et al., 2020). The genomic differences might be proved detrimental to important fitness traits as has been shown in both fruit flies and mosquitoes (Meza et al., 2011; Facchinelli et al., 2013; Rempoulakis et al., 2016; Ramírez-Santos et al., 2017; Ramírez-Santos et al., 2017; Carvalho et al., 2020). An in-depth quality control analysis that will assess important parameters like fecundity, fertility, longevity, flight ability, male mating competitiveness and response to irradiation, prior to upscaling and releasing in the field.

The release of a mosquito GSS as part of an operational SIT programme is ruled in most cases by concerns regarding the biosafety and biosecurity of the released strain, as well as by uncertainties related to the performance of the strain in the wild. A mosquito GSS has been developed and reared in laboratory conditions for several generations and carries its own genomic background. Decision-making bodies could reject the release of a GSS in an area based on the notion that breeding of human disease vectors bearing different genomic backgrounds could result in previously undetected risks related to humans and the environment. To address these issues, it is advisable to use either a local strain or to integrate the mosquito strains into the local genomic background prior to release. That way the potential effects associated with mating incompatibility are minimized and the chances for increased male mating competitiveness are raised since the released males and the wild females will share the same genomic background. In addition, maintaining the local genomic background can resolve any regulatory issues posed by the countries, reaffirm the biosecurity and biosafety of the released strain, and enhance the public acceptance towards the SIT programmes.

The recent discovery of the gene responsible for the red eye phenotype in *Aedes aegypti*, namely *cardinal*, opens the way for a faster and easier transfer of the sexing characters of the red eye GSS in local genomic background and will thus avoid the long (10–11 generations) and tedious genetic crosses described in this manuscript (Chen et al., 2021). This can be achieved by using CRISPR/Cas9 targeted mutagenesis of the *cardinal* gene of the local population to *de novo* develop a red eye mutant line. Next step would be to perform two simple genetic crosses as the ones described for the original construction of the red-eye GSS (Koskinioti et al., 2020). First, mutant females should be crossed with wild type males and second, F1 males should be backcrossed with mutant females to establish a genetic sexing strain with local genomic background.

## Data Availability

The original contributions presented in the study are included in the article/Supplementary Material, further inquiries can be directed to the corresponding author.

## References

[B1] Achee N. L. Gould F. Perkins T. A. Reiner R. C. Jr Morrison A. C. Ritchie S. A. (2015). A Critical Assessment of Vector Control for Dengue Prevention. Plos Negl. Trop. Dis. 9 (5), e0003655. 10.1371/journal.pntd.0003655 25951103 PMC4423954

[B2] Alphey L. McKemey A. Nimmo D. Neira Oviedo M. Lacroix R. Matzen K. (2013). Genetic Control ofAedesmosquitoes. Pathog. Glob. Health 107 (4), 170–179. 10.1179/2047773213Y.0000000095 23816508 PMC4001467

[B3] Aryan A. Anderson M. A. E. Biedler J. K. Qi Y. Overcash J. M. Naumenko A. N. (2020). Nixalone Is Sufficient to Convert femaleAedes Aegyptiinto fertile Males Andmyo-Sexis Needed for Male Flight. Proc. Natl. Acad. Sci. USA 117 (30), 17702–17709. 10.1073/pnas.2001132117 32661163 PMC7395513

[B4] Augustinos A. A. Misbah-ul-Haq M. Carvalho D. O. de la Fuente L. D. Koskinioti P. Bourtzis K. (2020). Irradiation Induced Inversions Suppress Recombination between the M Locus and Morphological Markers in *Aedes aegypti* . BMC Genet. 21, 142. 10.1186/s12863-020-00949-w 33339503 PMC7747368

[B5] Augustinos A. A. Targovska A. Cancio-Martinez E. Schorn E. Franz G. Cáceres C. (2017). Ceratitis Capitata Genetic Sexing Strains: Laboratory Evaluation of Strains from Mass-Rearing Facilities Worldwide. Entomol. Exp. Appl. 164, 305–317. 10.1111/eea.12612

[B6] Axford J. K. Callahan A. G. Hoffmann A. A. Yeap H. L. Ross P. A. (2016). Fitness of wAlbB Wolbachia Infection in *Aedes aegypti*: Parameter Estimates in an Outcrossed Background and Potential for Population Invasion. Am. J. Trop. Med. Hyg. 94 (3), 507–516. 10.4269/ajtmh.15-0608 26711515 PMC4775882

[B7] Beckham J. D. Tyler K. L. (2015). Arbovirus Infections. CONTINUUM: Lifelong Learn. Neurol. 21 (6 Neuroinfectious Disease), 1599–1611. 10.1212/CON.0000000000000240 PMC5089063 26633778

[B8] Bellini R. Medici A. Puggioli A. Balestrino F. Carrieri M. (2013). Pilot Field Trials with *Aedes albopictus* Irradiated Sterile Males in Italian Urban Areas. Jnl. Med. Entom. 50 (2), 317–325. 10.1603/me12048 23540120

[B9] Bennett K. E. Farfan-Ale J. A. Fernandez-Salas I. Black W. C. Higgs S. Beaty B. J. (2002). Variation in Vector Competence for Dengue 2 Virus Among 24 Collections of *Aedes aegypti* from Mexico and the United States. Am. J. Trop. Med. Hyg. 67 (1), 85–92. 10.4269/ajtmh.2002.67.85 12363070

[B10] Bhalla S. C. Craig Jr. G. B. (1970). Linkage Analysis of Chromosome I of Aedes Aegypti. Can. J. Genet. Cytol. 12 (3), 425–435. 10.1139/g70-061 5516679

[B11] Bhalla S. C. (1973). Sex-linked Translocations, Semisterility and Linkage Alterations in the Mosquito *Aedes aegypti* . Can. J. Genet. Cytol. 15 (1), 9–20. 10.1139/g73-002 4735778

[B12] Bhatt S. Gething P. W. Brady O. J. Messina J. P. Farlow A. W. Moyes C. L. (2013). The Global Distribution and burden of Dengue. Nature 496, 504–507. 10.1038/nature12060 23563266 PMC3651993

[B13] Bourtzis K. Dobson S. L. Xi Z. Rasgon J. L. Calvitti M. Moreira L. A. (2014). Harnessing Mosquito-Wolbachia Symbiosis for Vector and Disease Control. Acta Tropica 132 (Suppl. l), S150–S163. 10.1016/j.actatropica.2013.11.004 24252486

[B14] Bourtzis K. Lees R. S. Hendrichs J. Vreysen M. J. B. (2016). More Than One Rabbit Out of the Hat: Radiation, Transgenic and Symbiont-Based Approaches for Sustainable Management of Mosquito and Tsetse Fly Populations. Acta Tropica 157, 115–130. 10.1016/j.actatropica.2016.01.009 26774684

[B15] Bushland R. C. Lindquist A. W. Knipling E. F. (1955). Eradication of Screw-Worms through Release of Sterilized Males. Science 122 (3163), 287–288. 10.1126/science.122.3163.287 17751225

[B16] Campbell C. L. Dickson L. B. Lozano-Fuentes S. Juneja P. Jiggins F. M. Black W. C. (2017). Alternative Patterns of Sex Chromosome Differentiation in *Aedes aegypti* (L). BMC Genomics 18, 943. 10.1186/s12864-017-4348-4 29202694 PMC5716240

[B17] Carvalho D. O. McKemey A. R. Garziera L. Lacroix R. Donnelly C. A. Alphey L. (2015). Suppression of a Field Population of *Aedes aegypti* in Brazil by Sustained Release of Transgenic Male Mosquitoes. Plos Negl. Trop. Dis. 9 (7), e0003864. 10.1371/journal.pntd.0003864 26135160 PMC4489809

[B18] Carvalho D. O. Torres‐Monzon J. A. Koskinioti P. Dilrukshi Wijegunawardana N. D. A. Liang X. Pillwax G. (2020). *Aedes aegypti* Lines for Combined Sterile Insect Technique and Incompatible Insect Technique Applications: the Importance of Host Genomic Background. Entomol. Exp. Appl. 168, 560–572. 10.1111/eea.12892

[B19] Chen C. Compton A. Nikolouli K. Wang A. Aryan A. Sharma A. (2021). Marker-assisted Mapping Enables Effective Forward Genetic Analysis in the Arboviral Vector *Aedes aegypti*, a Species with Vast Recombination Deserts. bioRxiv. 10.1101/2021.04.29.442065 PMC9630976 36083009

[B20] Craig G. B. Jr Hickey W. A. (1967). Current Status of the Formal Genetics of *Aedes aegypti* . Bull. World Health Organ. 36 (4), 559–562. 5299452 PMC2476422

[B21] Crawford J. E. Clarke D. W. Criswell V. Desnoyer M. Cornel D. Deegan B. (2020). Efficient Production of Male Wolbachia-Infected *Aedes aegypti* Mosquitoes Enables Large-Scale Suppression of Wild Populations. Nat. Biotechnol. 38, 482–492. 10.1038/s41587-020-0471-x 32265562

[B22] Demétrio C. G. B. Hinde J. Moral R. A. (2014). “Models for Overdispersed Data in Entomology,” in Ecological Modelling Applied to Entomology. Entomology in Focus. Editors Ferreira, C. Godoy W. (Cham: Springer), Vol. 1, 219–259. 10.1007/978-3-319-06877-0_9

[B23] Dickson L. B. Sharakhova M. V. Timoshevskiy V. A. Fleming K. L. Caspary A. Sylla M. (2016). Reproductive Incompatibility Involving Senegalese *Aedes aegypti* (L) Is Associated with Chromosome Rearrangements. Plos Negl. Trop. Dis. 10 (4), e0004626. 10.1371/journal.pntd.0004626 27105225 PMC4841568

[B24] Dunn P. K. Smyth G. K. (2018). Generalized Linear Models with Examples in R. Springer Texts in Statistics. 1st ed. New York: Springer. ISBN 978-1-4419-0118-7.

[B25] Enkerlin W. R. (2021). “Impact of Fruit Fly Control Programmes Using the Sterile Insect Technique,” in Sterile Insect Technique Principles and Practice in Area-wide Integrated Pest Management. Editors Dyck V. A. Hendrichs J. Robinson A. S. (New York: CRC Press), 979–1006. 10.1201/9781003035572-30

[B26] Facchinelli L. Valerio L. Ramsey J. M. Gould F. WalshBond R. K. G. Bond G. (2013). Field Cage Studies and Progressive Evaluation of Genetically-Engineered Mosquitoes. Plos Negl. Trop. Dis. 7 (1), e2001. 10.1371/journal.pntd.0002001 23350003 PMC3547837

[B27] Flores H. A. O’Neill S. L. (2018). Controlling Vector-Borne Diseases by Releasing Modified Mosquitoes. Nat. Rev. Microbiol. 16 (8), 508–518. 10.1038/s41579-018-0025-0 29777177 PMC7612058

[B28] Focks D. A. (1980). An Improved Separator for the Developmental Stages, Sexes, and Species of Mosquitoes (Diptera: Culicidae). J. Med. Entomol. 17 (6), 567–568. 10.1093/jmedent/17.6.567 6111610

[B29] Franz G. Bourtzis K. Cáceres C. (2021). “Practical and Operational Genetic Sexing Systems Based on Classical Genetic Approaches in Fruit Flies, an Example for Other Species Amenable to Large-Scale Rearing for the Sterile Insect Technique,” in ” in Sterile Insect Technique Principles and Practice in Area-wide Integrated Pest Management. Editors Dyck V. A. Hendrichs J. Robinson A. S. (New York: CRC Press), 575–604. 10.1201/9781003035572-17

[B30] Gilles J. R. L. Schetelig M. F. Scolari F. Marec F. Capurro M. L. Franz G. (2014). Towards Mosquito Sterile Insect Technique Programmes: Exploring Genetic, Molecular, Mechanical and Behavioural Methods of Sex Separation in Mosquitoes. Acta Tropica 132 (Suppl. l), S178–S187. 10.1016/j.actatropica.2013.08.015 23994521

[B31] Gunathilaka N. Ranathunge T. Udayanga L. Wijegunawardena A. Gilles J. R. L. Abeyewickreme W. (2019). Use of Mechanical and Behavioural Methods to Eliminate Female *Aedes aegypti* and *Aedes albopictus* for Sterile Insect Technique and Incompatible Insect Technique Applications. Parasites Vectors 12, 148. 10.1186/s13071-019-3398-7 30922368 PMC6437921

[B32] Hall A. B. Basu S. Jiang X. Qi Y. Timoshevskiy V. A. Biedler J. K. (2015). A Male-Determining Factor in the Mosquito *Aedes aegypti* . Science 348 (6240), 1268–1270. 10.1126/science.aaa2850 25999371 PMC5026532

[B33] Harris A. F. McKemey A. R. Nimmo D. Curtis Z. Black I. Morgan S. A. (2012). Successful Suppression of a Field Mosquito Population by Sustained Release of Engineered Male Mosquitoes. Nat. Biotechnol. 30, 828–830. 10.1038/nbt.2350 22965050

[B34] Iwamura T. Guzman-Holst A. Murray K. A. (2020). Accelerating Invasion Potential of Disease Vector *Aedes aegypti* under Climate Change. Nat. Commun. 11, 2130. 10.1038/s41467-020-16010-4 32358588 PMC7195482

[B35] Kandul N. P. Liu J. Sanchez C. H. M. Wu S. L. Marshall J. M. Akbari O. S. (2019). Transforming Insect Population Control with Precision Guided Sterile Males with Demonstration in Flies. Nat. Commun. 10, 84. 10.1038/s41467-018-07964-7 30622266 PMC6325135

[B36] Kittayapong P. Kaeothaisong No. Ninphanomchai S. Limohpasmanee W. (2018). Combined Sterile Insect Technique and Incompatible Insect Technique: Sex Separation and Quality of Sterile *Aedes aegypti* Male Mosquitoes Released in a Pilot Population Suppression Trial in Thailand. Parasites Vectors 11, 657. 10.1186/s13071-018-3214-9 30583749 PMC6304762

[B37] Kittayapong P. Ninphanomchai S. Limohpasmanee W. Chansang C. Chansang U. Mongkalangoon P. (2019). Combined Sterile Insect Technique and Incompatible Insect Technique: The First Proof-Of-Concept to Suppress *Aedes aegypti* Vector Populations in Semi-rural Settings in Thailand. PLOS Negl. Trop. Dis. 13 (10), e0007771. 10.1371/journal.pntd.0007771 31658265 PMC6837763

[B38] Klassen W. Curtis C. F. Hendrichs J. (2021). “History of the Sterile Insect Technique,” in ” in Sterile Insect Technique Principles and Practice in Area-wide Integrated Pest Management. Editors Dyck V. A. Hendrichs J. Robinson A. S. (New York: CRC Press), 1–44. 10.1201/9781003035572-1

[B39] Knipling E. F. (1955). Possibilities of Insect Control or Eradication through the Use of Sexually Sterile Males. J. Econ. Entomol. 48 (4), 459–462. 10.1093/jee/48.4.459

[B40] Koskinioti P. Augustinos A. A. Carvalho D. O. Misbah-ul-Haq M. Pillwax G. de la Fuente L. D. (2020). Genetic Sexing Strains for the Population Suppression of the Mosquito Vector *Aedes aegypti* . Philos. Trans. R. Soc. 376, 20190808. 10.1098/rstb.2019.0808 PMC7776939 33357054

[B41] Kraemer M. U. Sinka M. E. Duda K. A. Mylne A. Q. Shearer F. M. Barker C. M. (2015). The Global Distribution of the Arbovirus Vectors *Aedes aegypti* and Ae. Albopictus. Elife. 4, e08347. 10.7554/eLife.08347 26126267 PMC4493616

[B42] Krafsur E. S. Ouma J. O. (2021). “Role of Population Genetics in the Sterile Insect Technique,” in ” in Sterile Insect Technique Principles and Practice in Area-wide Integrated Pest Management. Editors Dyck V. A. Hendrichs J. Robinson A. S. (New York: CRC Press), 529–548. 10.1201/9781003035572-15

[B43] Kyrou K. Hammond A. Galizi R. Kranjc N. Burt A. Beaghton A. K. (2018). A CRISPR–Cas9 Gene Drive Targeting Doublesex Causes Complete Population Suppression in Caged *Anopheles gambiae* Mosquitoes. Nat. Biotechnol. 36, 1062–1066. 10.1038/nbt.4245 30247490 PMC6871539

[B44] Lees R. S. Gilles J. R. Hendrichs J. Vreysen M. J. Bourtzis K. (2015). Back to the Future: the Sterile Insect Technique against Mosquito Disease Vectors. Curr. Opin. Insect Sci. 10, 156–162. 10.1016/j.cois.2015.05.011 29588003

[B45] Lima E. P. Paiva M. H. S. de Araújo A. P. da Silva É. V. G. da Silva U. M. de Oliveira L. N. (2011). Insecticide Resistance in *Aedes aegypti* Populations from Ceará, Brazil. Parasites Vectors 4, 5. 10.1186/1756-3305-4-5 21226942 PMC3035027

[B46] Liu P. Jin B. Li X. Zhao Y. Gu J. Biedler J. K. (2020). Nix Is a Male-Determining Factor in the Asian Tiger Mosquito *Aedes albopictus* . Insect Biochem. Mol. Biol. 118, 103311. 10.1016/j.ibmb.2019.103311 31901476 PMC10211468

[B47] Louis V. R. Montenegro Quiñonez C. A. Kusumawathie P. Palihawadana P. Janaki S. Tozan Y. (2016). Characteristics of and Factors Associated with Dengue Vector Breeding Sites in the City of Colombo, Sri Lanka. Pathog. Glob. Health 110 (2), 79–86. 10.1080/20477724.2016.1175158 27241954 PMC4894263

[B48] Lucey D. R. Gostin L. O. (2016). The Emerging Zika Pandemic: Enhancing Preparedness. JAMA 315 (9), 865–866. 10.1001/jama.2016.0904 26818622

[B49] Mains J. Brelsfoard C. Rose R. Dobson S. L. (2016). Female Adult *Aedes albopictus* Suppression by Wolbachia-Infected Male Mosquitoes. Sci. Rep. 6, 33846. 10.1038/srep33846 27659038 PMC5034338

[B50] Menge D. M. Guda T. Zhong D. Pai A. Zhou G. Beier J. C. (2005). Fitness Consequences of *Anopheles gambiae* Population Hybridization. Malar. J. 4, 44. 10.1186/1475-2875-4-44 16174295 PMC1242248

[B51] Meza J. S. Nirmala X. Zimowska G. J. Zepeda-Cisneros C. S. Handler A. M. (2011). Development of Transgenic Strains for the Biological Control of the Mexican Fruit Fly. Anastrepha Ludens. Genetica. 139 (1), 53–62. 10.1007/s10709-010-9484-6 20737195

[B52] Moral R. D. Hinde J. P. Demétrio C. G. (2017). Half-Normal Plots and Overdispersed Models in R: The Hnp Package. J. Stat. Softw. 81, 1–23. 10.18637/jss.v081.i10

[B53] Moyes C. L. Vontas J. Martins A. J. Ng L. C. Koou S. Y. Dusfour I. (2017). Contemporary Status of Insecticide Resistance in the Major Aedes Vectors of Arboviruses Infecting Humans. Plos Negl. Trop. Dis. 11 (7), e0005625. 10.1371/journal.pntd.0005625 28727779 PMC5518996

[B54] Munstermann L. E. Craig G. B. (1979). Genetics of *Aedes aegypti*: Updating the Linkage Map. J. Hered. 70 (5), 291–296. 10.1093/oxfordjournals.jhered.a109261

[B55] Nelder J. A. Wedderburn R. W. M. (1972). Generalized Linear Models. J. R. Stat. Soc. Ser. A (General) 135 (3), 370–384. 10.2307/2344614

[B56] Newton M. E. Southern D. I. Wood R. J. (1974). X and Y Chromosomes of Aedes Aegypti (L.) Distinguished by Giemsa C-Banding. Chromosoma 49 (1). 10.1007/BF00284986 4141301

[B57] O'Connor L. Plichart C. Sang A. C. Brelsfoard C. L. Bossin H. C. Dobson S. L. (2012). Open Release of Male Mosquitoes Infected with a Wolbachia Biopesticide: Field Performance and Infection Containment. PLOS Negl. Trop. Dis. 6 (11), e1797. 10.1371/journal.pntd.0001797 23166845 PMC3499408

[B58] Paixão E. S. Teixeira M. G. Rodrigues L. C. (2018). Zika, Chikungunya and Dengue: the Causes and Threats of New and Re-emerging Arboviral Diseases. BMJ Glob. Health 3 (Suppl. 1), e000530. 10.1136/bmjgh-2017-000530 PMC5759716 29435366

[B59] Papathanos P. A. Bossin H. C. Benedict M. Q. Catteruccia F. Malcolm C. A. Alphey L. (2009). Sex Separation Strategies: Past Experience and New Approaches. Malar. J. 8, S5. 10.1186/1475-2875-8-S2-S5 PMC2777327 19917075

[B60] Papathanos P. A. Bourtzis K. Tripet F. Bossin H. Virginio J. F. Capurro M. L. (2018). A Perspective on the Need and Current Status of Efficient Sex Separation Methods for Mosquito Genetic Control. Parasites Vectors 11, 654. 10.1186/s13071-018-3222-9 30583720 PMC6304774

[B61] R Core Team (2021). R: A Language and Environment for Statistical Computing. https://www.R-project.org/.

[B62] Ramírez-Santos E. M. Rendón P. Ruiz-Montoya L. Toledo J. Liedo P. (2017a). Performance of a Genetically Modified Strain of the Mediterranean Fruit Fly (Diptera: Tephritidae) for Area-wide Integrated Pest Management with the Sterile Insect Technique. J. Econ. Entomol. 110 (1), 24–34. 10.1093/jee/tow239 28011689

[B63] Ramírez-Santos E. Rendón P. Ruiz-Montoya L. Toledo J. Liedo P. (2017b). Effect of Irradiation Doses on Sterility and Biological Security in a Genetically Modified Strain of the Mediterranean Fruit Fly (Diptera: Tephritidae). J. Econ. Entomol. 110 (4), 1483–1494. 10.1093/jee/tox119 28854644

[B64] Rempoulakis P. Taret G. Haq I. Wornayporn V. Ahmad S. Tomas U. S. (2016). Evaluation of Quality Production Parameters and Mating Behavior of Novel Genetic Sexing Strains of the Mediterranean Fruit Fly Ceratitis Capitata (Wiedemann) (Diptera: Tephritidae). PLOS ONE 11 (6), e0157679. 10.1371/journal.pone.0157679 27336737 PMC4918918

[B65] Ryan S. J. Carlson C. J. Mordecai E. A. Johnson L. R. (2019). Global Expansion and Redistribution of Aedes-Borne Virus Transmission Risk with Climate Change. Plos Negl. Trop. Dis. 13 (3), e0007213. 10.1371/journal.pntd.0007213 30921321 PMC6438455

[B66] Scott T. W. Takken W. (2012). Feeding Strategies of Anthropophilic Mosquitoes Result in Increased Risk of Pathogen Transmission. Trends Parasitol. 28 (3), 114–121. 10.1016/j.pt.2012.01.001 22300806

[B67] Searle S. R. Speed F. M. Milliken G. A. (1980). Population Marginal Means in the Linear Model: An Alternative to Least Squares Means. The Am. Statistician 34 (4), 216–221. 10.2307/2684063

[B68] Siraj A. S. Oidtman R. J. Huber J. H. Kraemer M. Brady O. J. Johansson M. A. (2017). Temperature Modulates Dengue Virus Epidemic Growth Rates through its Effects on Reproduction Numbers and Generation Intervals. Plos Negl. Trop. Dis. 11 (7), e0005797. 10.1371/journal.pntd.0005797 28723920 PMC5536440

[B69] Struchiner C. J. Rocklöv J. Wilder-Smith A. Massad E. (2015). Increasing Dengue Incidence in Singapore over the Past 40 years: Population Growth, Climate and Mobility. PLOS ONE 10 (8), e0136286. 10.1371/journal.pone.0136286 26322517 PMC4554991

[B70] Timoshevskiy V. A. Severson D. W. Debruyn B. S. Black W. C. Sharakhov I. V. Sharakhova M. V. (2013). An Integrated Linkage, Chromosome, and Genome Map for the Yellow Fever Mosquito *Aedes aegypti* . Plos Negl. Trop. Dis. 7 (2), e2052. 10.1371/journal.pntd.0002052 23459230 PMC3573077

[B71] Weaver S. C. Reisen W. K. (2010). Present and Future Arboviral Threats. Antivir. Res 85 (2), 328–345. 10.1016/j.antiviral.2009.10.008 19857523 PMC2815176

[B72] Wilder-Smith A. Gubler D. J. (2008). Geographic Expansion of Dengue: the Impact of International Travel. Med. Clin. North. Am. 92 (6), 1377–1390. x. 10.1016/j.mcna.2008.07.002 19061757

[B73] Wilder-Smith A. Gubler D. J. Weaver S. C. Monath T. P. Heymann D. L. Scott T. W. (2017). Epidemic Arboviral Diseases: Priorities for Research and Public Health. Lancet Infect. Dis. 17 (3), e101–e106. 10.1016/S1473-3099(16)30518-7 28011234

[B74] Zacharopoulou A. Augustinos A. Drosopoulou E. Tsoumani K. Gariou-Papalexiou A. Franz G. (2017). A Review of More Than 30 Years of Cytogenetic Studies of Tephritidae in Support of Sterile Insect Technique and Global Trade. Entomol. Exp. Appl. 164 (3), 204–225. 10.1111/eea.12616

[B75] Zheng X. Zhang D. Li Y. Yang C. Wu Y. Liang X. (2019). Incompatible and Sterile Insect Techniques Combined Eliminate Mosquitoes. Nature 572, 56–61. 10.1038/s41586-019-1407-9 31316207

