# Supplementary material for: Introgression of the Aedes aegypti Red-Eye Genetic Sexing Strains Into Different Genomic Backgrounds for Sterile Insect Technique Applications
Source: Front Bioeng Biotechnol. 2022 Feb 2;10:821428. doi: 10.3389/fbioe.2022.821428 (PMC8847382; doi:10.3389/fbioe.2022.821428)
Supplement: Supplementary file 1 [file Table1.DOCX]

Supplementary Material

# Supplementary Material 1

**Crossing scheme to get a Red-eye GSS highly introgressed into a local genomic background**

*Abbreviations*

- re: the mutated allele for the red eye colour
- re^+^: the wild type allele for the black eye colour
- re^+^ > re: the wild type re^+^ is dominant over the re. That means that re^+^/re^+^ and re^+^/re genotypes are wild type (black), while only re/re genotypes are red
- The red eye locus is located on chromosome 1 of *Ae. aegypti* and is linked to the sex determining M locus. Therefore:
- re m / re m = red eye females
- re^+^ m / re m = black eye females
- re m / re^+^ m = black eye females
- re^+^ m / re^+^ m = black eye females
- re M / re m = red eye males
- re^+^ M / re m = black eye males
- re M / re^+^ m = black eye males
- re^+^ M / re^+^ m = black eye males

*Experimental approach*

- F0 (parental cross):
  - Re m/re m (red eye strain) x re^+^ M/re^+^ m (“wild type” strain) (P).
  - *F1 progeny are all wild type and with about 50% “wild type” genomic background.*
- F1:
  - Backcross F1 females with “wild type” males.
  - *F2 progeny are all wild type and with about 75% “wild type” genomic background.*
- F2:
  - Inbreed F2 males and females.
  - *F3 progeny have mainly black eyes but some are expected to have the red eye phenotype (mainly females). At the same time, they are considered to have about 75% “wild type” genomic background.*
- F3:
  - Backcross F3 red eye females with “wild type” males.
  - *F4 progeny are all wild type and with about 87.5 % “wild type” genomic background.*
- F4:
  - Backcross F4 females with “wild type” males.
  - *F5 progeny are all wild type and with about 93.625% “wild type” genomic background.*
- F5:
- Inbreed F5 males and females.
- *F6 progeny have mainly black eyes but some are expected to have the red eye phenotype (mainly females). At the same time, they are considered to have about 93.625 % “wild type” genomic background.*
- F6:
  - Backcross F6 red eye females with “wild type” males.
  - *F7 progeny are all wild type and with about 96.8% “wild type” genomic background.*
- F7:
  - Backcross F7 females with “wild type” males.
  - *F8 progeny are all wild type and with about 98.44% “wild type” genomic background.*
- F8:
  - Inbreed F8 males and females.
  - *F9 progeny have mainly black eyes but some are expected to have the red eye phenotype (mainly females). At the same time, they are considered to have about 98.44 % “wild type” genomic background.*
- F9:
- Backcross F9 red eye females with “wild type” males.

*F10a progeny are all wild type and with about 99.21 % “wild type” genomic background.*

- At the same time, inbreed F9 red eye females and red eye males (red eye males are very rare).

*This red eye line (F10b) can be considered as having about 98.44% “wild type” genomic background.*

- F10:
  - Cross F10b red eye females with F10a wild type males. This is a GSS that is considered to have a “wild type” genomic background of *about* 98.82 %.
