# Supplementary material for: Introgression of the Aedes aegypti Red-Eye Genetic Sexing Strains Into Different Genomic Backgrounds for Sterile Insect Technique Applications
Source: Front Bioeng Biotechnol. 2022 Feb 2;10:821428. doi: 10.3389/fbioe.2022.821428 (PMC8847382; doi:10.3389/fbioe.2022.821428)
Supplement: Supplementary file 2 [file Table7.DOCX]

Supplementary Material

# Supplementary Material 7

####Pairwise comparisons of generations per strain.

#BRA Red-Eye GSS

contrast estimate SE df z.ratio p.value

1 - 2 -0.1805 0.333 Inf -0.542 0.5878

1 - 3 -0.1463 0.281 Inf -0.520 0.6031

1 - 4 -0.3760 0.306 Inf -1.229 0.2189

1 - 7 -0.2421 0.283 Inf -0.856 0.3922

1 - 8 0.2635 0.277 Inf 0.950 0.3419

2 - 3 0.0342 0.297 Inf 0.115 0.9085

2 - 4 -0.1955 0.321 Inf -0.610 0.5418

2 - 7 -0.0616 0.299 Inf -0.206 0.8366

2 - 8 0.4440 0.293 Inf 1.514 0.1301

3 - 4 -0.2297 0.267 Inf -0.862 0.3887

3 - 7 -0.0958 0.240 Inf -0.399 0.6897

3 - 8 0.4098 0.233 Inf 1.758 0.0787

4 - 7 0.1339 0.268 Inf 0.500 0.6174

4 - 8 0.6395 0.262 Inf 2.440 0.0147

7 - 8 0.5056 0.235 Inf 2.152 0.0314

#BRA Red-Eye GSS/Inv35

contrast estimate SE df z.ratio p.value

1 - 2 0.6166 0.914 Inf 0.675 0.4998

1 - 3 nonEst NA NA NA NA

1 - 4 0.2767 1.156 Inf 0.239 0.8108

1 - 7 0.7465 0.817 Inf 0.913 0.3610

1 - 8 0.3520 0.731 Inf 0.481 0.6302

2 - 3 nonEst NA NA NA NA

2 - 4 -0.3399 1.226 Inf -0.277 0.7816

2 - 7 0.1299 0.914 Inf 0.142 0.8869

2 - 8 -0.2646 0.837 Inf -0.316 0.7520

3 - 4 nonEst NA NA NA NA

3 - 7 nonEst NA NA NA NA

3 - 8 nonEst NA NA NA NA

4 - 7 0.4698 1.156 Inf 0.406 0.6844

4 - 8 0.0753 1.097 Inf 0.069 0.9452

7 - 8 -0.3945 0.731 Inf -0.540 0.5893

#IDN Red-Eye GSS

contrast estimate SE df z.ratio p.value

1 - 2 0.312405 0.202 Inf 1.548 0.1216

1 - 3 0.346710 0.194 Inf 1.783 0.0746

1 - 4 0.788705 0.212 Inf 3.722 0.0002

1 - 5 0.657761 0.197 Inf 3.344 0.0008

1 - 7 0.595670 0.208 Inf 2.861 0.0042

1 - 8 0.594842 0.212 Inf 2.803 0.0051

2 - 3 0.034305 0.221 Inf 0.155 0.8765

2 - 4 0.476300 0.236 Inf 2.016 0.0438

2 - 5 0.345356 0.223 Inf 1.551 0.1209

2 - 7 0.283265 0.233 Inf 1.216 0.2239

2 - 8 0.282436 0.236 Inf 1.194 0.2324

3 - 4 0.441995 0.230 Inf 1.922 0.0547

3 - 5 0.311051 0.216 Inf 1.439 0.1500

3 - 7 0.248960 0.227 Inf 1.099 0.2720

3 - 8 0.248131 0.230 Inf 1.077 0.2813

4 - 5 -0.130944 0.232 Inf -0.565 0.5723

4 - 7 -0.193034 0.242 Inf -0.798 0.4246

4 - 8 -0.193863 0.245 Inf -0.791 0.4291

5 - 7 -0.062091 0.229 Inf -0.272 0.7859

5 - 8 -0.062920 0.232 Inf -0.271 0.7864

7 - 8 -0.000829 0.242 Inf -0.003 0.9973

#IDN Red-Eye GSS/Inv35

contrast estimate SE df z.ratio p.value

1 - 2 -0.76285 1.051 Inf -0.726 0.4678

1 - 3 -0.00596 1.097 Inf -0.005 0.9957

1 - 4 0.30364 1.120 Inf 0.271 0.7863

1 - 5 0.02445 1.082 Inf 0.023 0.9820

1 - 7 -0.25557 1.082 Inf -0.236 0.8133

1 - 8 0.01246 1.097 Inf 0.011 0.9909

2 - 3 0.75689 0.549 Inf 1.379 0.1680

2 - 4 1.06649 0.593 Inf 1.799 0.0720

2 - 5 0.78730 0.518 Inf 1.521 0.1282

2 - 7 0.50728 0.518 Inf 0.980 0.3272

2 - 8 0.77531 0.549 Inf 1.412 0.1579

3 - 4 0.30960 0.672 Inf 0.461 0.6449

3 - 5 0.03041 0.607 Inf 0.050 0.9600

3 - 7 -0.24961 0.607 Inf -0.411 0.6807

3 - 8 0.01842 0.633 Inf 0.029 0.9768

4 - 5 -0.27919 0.646 Inf -0.432 0.6658

4 - 7 -0.55921 0.647 Inf -0.865 0.3871

4 - 8 -0.29118 0.672 Inf -0.433 0.6647

5 - 7 -0.28002 0.578 Inf -0.484 0.6283

5 - 8 -0.01199 0.607 Inf -0.020 0.9842

7 - 8 0.26803 0.607 Inf 0.442 0.6586

#LKA Red-Eye GSS

contrast estimate SE df z.ratio p.value

1 - 2 1.2096 0.321 Inf 3.764 0.0002

1 - 3 1.3750 0.311 Inf 4.422 <.0001

1 - 4 0.5581 0.238 Inf 2.340 0.0193

1 - 7 0.6998 0.260 Inf 2.693 0.0071

1 - 8 0.7630 0.218 Inf 3.507 0.0005

2 - 3 0.1654 0.402 Inf 0.411 0.6809

2 - 4 -0.6515 0.349 Inf -1.866 0.0620

2 - 7 -0.5098 0.364 Inf -1.400 0.1614

2 - 8 -0.4466 0.335 Inf -1.332 0.1827

3 - 4 -0.8169 0.340 Inf -2.406 0.0161

3 - 7 -0.6752 0.355 Inf -1.903 0.0571

3 - 8 -0.6120 0.325 Inf -1.882 0.0599

4 - 7 0.1417 0.293 Inf 0.483 0.6293

4 - 8 0.2049 0.257 Inf 0.798 0.4250

7 - 8 0.0632 0.277 Inf 0.228 0.8193

#LKA Red-Eye GSS/Inv35

contrast estimate SE df z.ratio p.value

1 - 2 -24.568 8.47e+04 Inf 0.000 0.9998

1 - 3 -22.856 8.47e+04 Inf 0.000 0.9998

1 - 4 -23.958 8.47e+04 Inf 0.000 0.9998

1 - 7 0.834 1.60e+06 Inf 0.000 1.0000

1 - 8 -25.031 8.47e+04 Inf 0.000 0.9998

2 - 3 1.712 1.10e+00 Inf 1.562 0.1183

2 - 4 0.610 8.00e-01 Inf 0.729 0.4662

2 - 7 25.402 1.60e+06 Inf 0.000 1.0000

2 - 8 -0.462 6.00e-01 Inf -0.762 0.4461

3 - 4 -1.102 1.20e+00 Inf -0.899 0.3686

3 - 7 23.690 1.60e+06 Inf 0.000 1.0000

3 - 8 -2.174 1.10e+00 Inf -2.012 0.0442

4 - 7 24.792 1.60e+06 Inf 0.000 1.0000

4 - 8 -1.073 8.00e-01 Inf -1.312 0.1895

7 - 8 -25.864 1.60e+06 Inf 0.000 1.0000

#MEX Red-Eye GSS

contrast estimate SE df z.ratio p.value

1 - 2 -0.2135 0.303 Inf -0.705 0.4811

1 - 3 -0.4209 0.281 Inf -1.498 0.1342

1 - 4 -0.2237 0.324 Inf -0.690 0.4903

1 - 5 -0.3535 0.312 Inf -1.133 0.2572

1 - 7 -0.0825 0.302 Inf -0.273 0.7845

1 - 8 -0.3314 0.269 Inf -1.232 0.2178

1 - 9 0.1772 0.290 Inf 0.612 0.5408

2 - 3 -0.2075 0.207 Inf -1.002 0.3162

2 - 4 -0.0103 0.263 Inf -0.039 0.9689

2 - 5 -0.1400 0.247 Inf -0.566 0.5713

2 - 7 0.1310 0.234 Inf 0.559 0.5758

2 - 8 -0.1179 0.190 Inf -0.620 0.5351

2 - 9 0.3907 0.219 Inf 1.787 0.0740

3 - 4 0.1972 0.237 Inf 0.832 0.4055

3 - 5 0.0674 0.220 Inf 0.307 0.7592

3 - 7 0.3384 0.205 Inf 1.651 0.0987

3 - 8 0.0895 0.153 Inf 0.586 0.5579

3 - 9 0.5982 0.187 Inf 3.197 0.0014

4 - 5 -0.1298 0.273 Inf -0.475 0.6345

4 - 7 0.1412 0.261 Inf 0.541 0.5886

4 - 8 -0.1077 0.223 Inf -0.484 0.6285

4 - 9 0.4010 0.247 Inf 1.621 0.1050

5 - 7 0.2710 0.246 Inf 1.103 0.2699

5 - 8 0.0221 0.204 Inf 0.108 0.9138

5 - 9 0.5308 0.231 Inf 2.298 0.0216

7 - 8 -0.2489 0.188 Inf -1.324 0.1854

7 - 9 0.2597 0.217 Inf 1.198 0.2308

8 - 9 0.5087 0.168 Inf 3.022 0.0025

#MEX Red-Eye GSS/Inv35

contrast estimate SE df z.ratio p.value

1 - 2 0.00649 0.708 Inf 0.009 0.9927

1 - 3 1.22408 1.119 Inf 1.094 0.2739

1 - 4 0.20528 0.867 Inf 0.237 0.8128

1 - 5 -0.07011 0.867 Inf -0.081 0.9356

1 - 7 -0.07291 0.708 Inf -0.103 0.9180

1 - 8 1.16168 0.867 Inf 1.340 0.1801

1 - 9 1.22714 0.867 Inf 1.416 0.1568

2 - 3 1.21759 1.119 Inf 1.088 0.2764

2 - 4 0.19879 0.867 Inf 0.229 0.8187

2 - 5 -0.07660 0.867 Inf -0.088 0.9296

2 - 7 -0.07940 0.708 Inf -0.112 0.9107

2 - 8 1.15520 0.867 Inf 1.333 0.1825

2 - 9 1.22065 0.867 Inf 1.409 0.1590

3 - 4 -1.01881 1.225 Inf -0.831 0.4058

3 - 5 -1.29419 1.226 Inf -1.056 0.2910

3 - 7 -1.29699 1.119 Inf -1.159 0.2463

3 - 8 -0.06240 1.225 Inf -0.051 0.9594

3 - 9 0.00306 1.225 Inf 0.002 0.9980

4 - 5 -0.27539 1.001 Inf -0.275 0.7833

4 - 7 -0.27818 0.867 Inf -0.321 0.7483

4 - 8 0.95641 1.001 Inf 0.956 0.3392

4 - 9 1.02186 1.001 Inf 1.021 0.3072

5 - 7 -0.00280 0.867 Inf -0.003 0.9974

5 - 8 1.23179 1.001 Inf 1.231 0.2184

5 - 9 1.29725 1.001 Inf 1.296 0.1949

7 - 8 1.23459 0.867 Inf 1.425 0.1543

7 - 9 1.30005 0.867 Inf 1.500 0.1336

8 - 9 0.06545 1.000 Inf 0.065 0.9478

#SGP Red-Eye GSS

contrast estimate SE df z.ratio p.value

1 - 2 0.0185 0.431 Inf 0.043 0.9657

1 - 3 -0.4823 0.337 Inf -1.430 0.1527

1 - 4 -0.5170 0.313 Inf -1.654 0.0982

2 - 3 -0.5008 0.393 Inf -1.276 0.2020

2 - 4 -0.5355 0.372 Inf -1.441 0.1495

3 - 4 -0.0347 0.257 Inf -0.135 0.8927

#SGP Red-Eye GSS/Inv35

contrast estimate SE df z.ratio p.value

1 - 2 -0.13 1.41 Inf -0.092 0.9268

##THA Red-Eye GSS

contrast estimate SE df z.ratio p.value

1 - 2 0.2685 0.294 Inf 0.912 0.3616

1 - 3 0.3803 0.300 Inf 1.267 0.2051

1 - 4 0.4527 0.342 Inf 1.322 0.1861

1 - 5 0.0264 0.284 Inf 0.093 0.9260

1 - 7 -0.0373 0.265 Inf -0.141 0.8882

1 - 8 0.2429 0.324 Inf 0.751 0.4529

2 - 3 0.1117 0.263 Inf 0.425 0.6707

2 - 4 0.1841 0.310 Inf 0.594 0.5528

2 - 5 -0.2422 0.244 Inf -0.991 0.3216

2 - 7 -0.3058 0.222 Inf -1.377 0.1685

2 - 8 -0.0256 0.289 Inf -0.089 0.9294

3 - 4 0.0724 0.316 Inf 0.229 0.8186

3 - 5 -0.3539 0.251 Inf -1.408 0.1590

3 - 7 -0.4176 0.230 Inf -1.818 0.0691

3 - 8 -0.1374 0.295 Inf -0.465 0.6416

4 - 5 -0.4263 0.300 Inf -1.419 0.1560

4 - 7 -0.4900 0.283 Inf -1.733 0.0830

4 - 8 -0.2098 0.338 Inf -0.621 0.5349

5 - 7 -0.0637 0.208 Inf -0.306 0.7599

5 - 8 0.2165 0.279 Inf 0.776 0.4375

7 - 8 0.2802 0.260 Inf 1.079 0.2804

##THA Red-Eye GSS/Inv35

contrast estimate SE df z.ratio p.value

1 - 2 0.242 0.91 Inf 0.265 0.7908

1 - 3 -0.396 0.80 Inf -0.493 0.6223

1 - 4 1.291 1.23 Inf 1.053 0.2922

1 - 6 1.172 1.23 Inf 0.957 0.3388

1 - 7 23.910 51382.37 Inf 0.000 0.9996

2 - 3 -0.638 0.69 Inf -0.923 0.3558

2 - 4 1.048 1.16 Inf 0.908 0.3641

2 - 6 0.930 1.16 Inf 0.805 0.4209

2 - 7 23.668 51382.37 Inf 0.000 0.9996

3 - 4 1.686 1.07 Inf 1.577 0.1149

3 - 6 1.568 1.07 Inf 1.466 0.1427

3 - 7 24.306 51382.37 Inf 0.000 0.9996

4 - 6 -0.119 1.41 Inf -0.084 0.9332

4 - 7 22.619 51382.37 Inf 0.000 0.9996

6 - 7 22.738 51382.37 Inf 0.000 0.9996
