# Supplementary material for: Introgression of the Aedes aegypti Red-Eye Genetic Sexing Strains Into Different Genomic Backgrounds for Sterile Insect Technique Applications
Source: Front Bioeng Biotechnol. 2022 Feb 2;10:821428. doi: 10.3389/fbioe.2022.821428 (PMC8847382; doi:10.3389/fbioe.2022.821428)
Supplement: Supplementary file 3 [file Table2.DOCX]

Supplementary Material

# Supplementary Material 2

**Crossing scheme to get a Red-eye GSS/Inv35 highly introgressed into a local genomic background**

*Abbreviations*

(see also Suppl. Material 1)

- Inv35: it is an inversion on the M chromosome that reduces recombination between the M locus and the red eye mutation (<0.3%).

*Experimental approach*

This crossing scheme is divided in three parts. Part 1A is identical to Suppl. Method 1 and it is performed to get highly introgressed red eye females. Part 1B is performed to get the Inv35 line partially introgressed into the local genomic background. In Part 2, the highly introgressed red eye females (Part 1A) are crossed with the partially introgressed Inv35 males (Part 1B).

**Part 1A:** Crossing scheme to get red eye females highly introgressed in the local genomic (follow Suppl. Method 1).

**Part 1B**

- Cross ‘wild type’ females with Inv35 males
- Backcross F1 males (black eye) with ‘wild type’ females
- Repeat the backcrossing for two additional times.
  - *This results to males with the Inv35 and ~97% ‘wild type’ genomic background.*

**Part 2**

- Cross highly introgressed red eye females from Part 1A with partially introgressed black eye males from Part 1B.
- Backcross male progeny (black eye) with highly introgressed red eye females.
  - *These crosses result in a Red-eye GSS with the Inv35 and ~98% ‘wild type’ background.*
