# Supplementary material for: Introgression of the Aedes aegypti Red-Eye Genetic Sexing Strains Into Different Genomic Backgrounds for Sterile Insect Technique Applications
Source: Front Bioeng Biotechnol. 2022 Feb 2;10:821428. doi: 10.3389/fbioe.2022.821428 (PMC8847382; doi:10.3389/fbioe.2022.821428)
Supplement: Supplementary file 4 [file Table5.DOCX]

Supplementary Material

# Supplementary Material 5

###Analysis of the effect of inversion Inv35 (per population). Recombination rates were analyzed per genomic background using a GLM-binomial or GLM-Quasi-Binomial family and a logit link function. Strain was a statistically significant factor for all genomic backgrounds.

BRA

1. Analysis of Deviance Table

Model: binomial, link: logit

Response: cbind(recombinants, progeny - recombinants)

Terms added sequentially (first to last)

Df Deviance Resid. Df Resid. Dev Pr(>Chi)

NULL 11 163.902

strain 1 150.41 10 13.492 < 2.2e-16 ***

---

Signif. codes: 0 ‘***’ 0.001 ‘**’ 0.01 ‘*’ 0.05 ‘.’ 0.1 ‘ ’ 1

2. Pairwise comparisons

contrast estimate SE df z.ratio p.value

RGSS - RGSS_35 2.47 0.278 Inf 8.861 <.0001

Results are given on the log odds ratio (not the response) scale.

IDN

1. Analysis of Deviance Table

Model: quasibinomial, link: logit

Response: cbind(recombinants, progeny - recombinants)

Terms added sequentially (first to last)

Df Deviance Resid. Df Resid. Dev F Pr(>F)

NULL 13 230.352

strain 1 205.03 12 25.324 90.072 6.282e-07 ***

---

Signif. codes: 0 ‘***’ 0.001 ‘**’ 0.01 ‘*’ 0.05 ‘.’ 0.1 ‘ ’ 1

2. Pairwise comparisons

contrast estimate SE df z.ratio p.value

RGSS - RGSS_35 1.99 0.264 Inf 7.544 <.0001

Results are given on the log odds ratio (not the response) scale.

LKA

1.Analysis of Deviance Table

Model: quasibinomial, link: logit

Response: cbind(recombinants, progeny - recombinants)

Terms added sequentially (first to last)

Df Deviance Resid. Df Resid. Dev F Pr(>F)

NULL 11 177.070

strain 1 128.03 10 49.035 25.763 0.0004808 ***

---

Signif. codes: 0 ‘***’ 0.001 ‘**’ 0.01 ‘*’ 0.05 ‘.’ 0.1 ‘ ’ 1

2. Pairwise comparisons

contrast estimate SE df z.ratio p.value

RGSS - RGSS_35 2.33 0.622 Inf 3.742 0.0002

Results are given on the log odds ratio (not the response) scale.

MEX

1. Analysis of Deviance Table

Model: quasibinomial, link: logit

Response: cbind(recombinants, progeny - recombinants)

Terms added sequentially (first to last)

Df Deviance Resid. Df Resid. Dev F Pr(>F)

NULL 15 398.36

strain 1 376.85 14 21.51 256.08 2.156e-10 ***

---

Signif. codes: 0 ‘***’ 0.001 ‘**’ 0.01 ‘*’ 0.05 ‘.’ 0.1 ‘ ’ 1

2. Pairwise comparisons

contrast estimate SE df z.ratio p.value

RGSS - RGSS_35 2.86 0.272 Inf 10.507 <.0001

Results are given on the log odds ratio (not the response) scale.

SGP

1. Analysis of Deviance Table

Model: binomial, link: logit

Response: cbind(recombinants, progeny - recombinants)

Terms added sequentially (first to last)

Df Deviance Resid. Df Resid. Dev Pr(>Chi)

NULL 5 113.928

strain 1 109.21 4 4.721 < 2.2e-16 ***

---

Signif. codes: 0 ‘***’ 0.001 ‘**’ 0.01 ‘*’ 0.05 ‘.’ 0.1 ‘ ’ 1

2. contrast estimate SE df z.ratio p.value

RGSS - RGSS_35 3.83 0.715 Inf 5.358 <.0001

Results are given on the log odds ratio (not the response) scale.

THAI

1. Analysis of Deviance Table

Model: quasibinomial, link: logit

Response: cbind(recombinants, progeny - recombinants)

Terms added sequentially (first to last)

Df Deviance Resid. Df Resid. Dev F Pr(>F)

NULL 11 167.087

strain 1 150.62 10 16.469 100.37 1.563e-06 ***

---

Signif. codes: 0 ‘***’ 0.001 ‘**’ 0.01 ‘*’ 0.05 ‘.’ 0.1 ‘ ’ 1

2. Pairwise comparisons

contrast estimate SE df z.ratio p.value

RGSS - RGSS_35 2.43 0.339 Inf 7.154 <.0001

Results are given on the log odds ratio (not the response) scale.
