# Supplementary material for: Introgression of the Aedes aegypti Red-Eye Genetic Sexing Strains Into Different Genomic Backgrounds for Sterile Insect Technique Applications
Source: Front Bioeng Biotechnol. 2022 Feb 2;10:821428. doi: 10.3389/fbioe.2022.821428 (PMC8847382; doi:10.3389/fbioe.2022.821428)
Supplement: Supplementary file 5 [file Table3.DOCX]

Supplementary Material

# Supplementary Material 3

Recombination rates between re and the M locus in six different genomic backgrounds of the Red-eye GSS strains. (wt = wild type, re = red eye)

| **Genomic background** | **F** | **Genotypes** | | | | | **Recombination rates** |
| --- | --- | --- | --- | --- | --- | --- | --- |
|  |  | **Parental** | | **Recombinant** | | **Total** |  |
|  |  | **wt males** | **re females** | **re males** | **wt females** |  |  |
| Brazil | F1 | 582 | 582 | 11 | 9 | 1184 | 0.016 |
|  | F2 | 364 | 462 | 11 | 6 | 843 | 0.020 |
|  | F3 | 961 | 849 | 23 | 13 | 1846 | 0.019 |
|  | F4 | 495 | 464 | 12 | 12 | 983 | 0.024 |
|  | F7 | 711 | 888 | 17 | 18 | 1634 | 0.021 |
|  | F8 | 1374 | 1580 | 20 | 19 | 2993 | 0.013 |
| Indonesia | F1 | 1123 | 475 | 45 | 25 | 1668 | 0.041 |
|  | F2 | 674 | 574 | 23 | 17 | 1288 | 0.031 |
|  | F3 | 760 | 693 | 23 | 22 | 1498 | 0.030 |
|  | F4 | 940 | 768 | 18 | 16 | 1742 | 0.019 |
|  | F5 | 913 | 982 | 17 | 26 | 1938 | 0.022 |
|  | F7 | 691 | 800 | 20 | 16 | 1527 | 0.023 |
|  | F8 | 684 | 723 | 11 | 23 | 1441 | 0.023 |
| Mexico | F1 | 443 | 231 | 13 | 3 | 690 | 0.023 |
|  | F2 | 647 | 612 | 19 | 18 | 1296 | 0.028 |
|  | F3 | 1334 | 574 | 55 | 14 | 1977 | 0.034 |
|  | F4 | 607 | 235 | 20 | 5 | 867 | 0.028 |
|  | F5 | 682 | 235 | 24 | 7 | 948 | 0.032 |
|  | F7 | 1030 | 444 | 23 | 15 | 1512 | 0.025 |
|  | F8 | 2983 | 767 | 97 | 27 | 3874 | 0.032 |
|  | F9 | 1764 | 801 | 39 | 12 | 2616 | 0.019 |
| Singapore | F1 | 417 | 403 | 8 | 6 | 834 | 0.016 |
|  | F2 | 306 | 231 | 4 | 5 | 546 | 0.016 |
|  | F3 | 547 | 357 | 12 | 13 | 929 | 0.026 |
|  | F4 | 627 | 805 | 20 | 21 | 1473 | 0.027 |
| Sri Lanka | F1 | 826 | 805 | 47 | 7 | 1685 | 0.032 |
|  | F2 | 840 | 375 | 9 | 3 | 1227 | 0.009 |
|  | F3 | 800 | 753 | 6 | 7 | 1566 | 0.008 |
|  | F4 | 645 | 780 | 12 | 15 | 1452 | 0.018 |
|  | F7 | 496 | 781 | 8 | 13 | 1298 | 0.016 |
|  | F8 | 1093 | 1239 | 16 | 20 | 2368 | 0.015 |
| Thailand | F1 | 473 | 369 | 6 | 13 | 861 | 0.022 |
|  | F2 | 1122 | 675 | 17 | 14 | 1828 | 0.016 |
|  | F3 | 1119 | 696 | 5 | 23 | 1843 | 0.015 |
|  | F4 | 598 | 517 | 6 | 10 | 1131 | 0.014 |
|  | F5 | 1046 | 683 | 21 | 17 | 1767 | 0.021 |
|  | F7 | 1394 | 1253 | 50 | 12 | 2709 | 0.022 |
|  | F8 | 526 | 604 | 11 | 9 | 1150 | 0.017 |
