# Supplementary material for: Introgression of the Aedes aegypti Red-Eye Genetic Sexing Strains Into Different Genomic Backgrounds for Sterile Insect Technique Applications
Source: Front Bioeng Biotechnol. 2022 Feb 2;10:821428. doi: 10.3389/fbioe.2022.821428 (PMC8847382; doi:10.3389/fbioe.2022.821428)
Supplement: Supplementary file 6 [file Table4.DOCX]

Supplementary Material

# Supplementary Material 4

Recombination rates between re and the M locus in six different genomic backgrounds of the Red-eye GSS/Inv35 strains (wt = wild type, re = red eye)

| **Genomic background** | **F** | **Genotypes** | | | | | **Recombination rates** |
| --- | --- | --- | --- | --- | --- | --- | --- |
|  |  | **Parental** | | **Recombinant** | | **Total** |  |
|  |  | **wt males** | **re females** | **re males** | **wt females** |  |  |
| Brazil | F1 | 576 | 500 | 3 | 0 | 1079 | 0.0027 |
|  | F2 | 697 | 632 | 2 | 0 | 1331 | 0.0015 |
|  | F3 | 674 | 611 | 0 | 0 | 1285 | 0 |
|  | F4 | 246 | 227 | 0 | 1 | 474 | 0.0021 |
|  | F7 | 1288 | 982 | 2 | 1 | 2273 | 0.0013 |
|  | F8 | 1274 | 1276 | 3 | 2 | 2555 | 0.0019 |
| Indonesia | F1 | 147 | 156 | 0 | 1 | 304 | 0.003 |
|  | F2 | 822 | 591 | 4 | 6 | 1423 | 0.007 |
|  | F3 | 714 | 792 | 2 | 3 | 1511 | 0.003 |
|  | F4 | 776 | 866 | 0 | 4 | 1646 | 0.002 |
|  | F5 | 1335 | 528 | 1 | 5 | 1869 | 0.003 |
|  | F7 | 765 | 643 | 3 | 3 | 1414 | 0.004 |
|  | F8 | 921 | 613 | 0 | 5 | 1539 | 0.003 |
| Mexico | F1 | 364 | 402 | 1 | 3 | 1540 | 0.0051 |
|  | F2 | 881 | 665 | 2 | 2 | 1550 | 0.0025 |
|  | F3 | 704 | 602 | 0 | 1 | 1307 | 0.0007 |
|  | F4 | 516 | 427 | 0 | 2 | 945 | 0.0021 |
|  | F5 | 392 | 324 | 1 | 1 | 718 | 0.0027 |
|  | F7 | 800 | 628 | 3 | 1 | 1432 | 0.0027 |
|  | F8 | 1242 | 1212 | 1 | 1 | 2456 | 0.0008 |
|  | F9 | 1307 | 1313 | 1 | 1 | 2622 | 0.0007 |
| Singapore | F1 | 998 | 1044 | 0 | 1 | 2043 | 0.0004 |
|  | F2 | 952 | 841 | 1 | 0 | 1794 | 0.0005 |
| Sri Lanka | F1 | 394 | 373 | 0 | 0 | 767 | 0 |
|  | F2 | 662 | 939 | 0 | 5 | 1606 | 0.003 |
|  | F3 | 676 | 1098 | 0 | 1 | 1775 | 0.0005 |
|  | F4 | 588 | 591 | 1 | 1 | 1181 | 0.0016 |
|  | F7 | 1023 | 744 | 0 | 0 | 1767 | 0 |
|  | F8 | 507 | 703 | 4 | 2 | 1216 | 0.004 |
| Thailand | F1 | 417 | 385 | 2 | 0 | 804 | 0.002 |
|  | F2 | 853 | 680 | 1 | 2 | 1536 | 0.0019 |
|  | F3 | 963 | 927 | 0 | 7 | 1897 | 0.003 |
|  | F4 | 697 | 761 | 0 | 1 | 1459 | 0.0006 |
|  | F6 | 630 | 665 | 0 | 1 | 1296 | 0.0007 |
|  | F7 | 582 | 771 | 0 | 0 | 1353 | 0 |
