# Supplementary material for: Introgression of the Aedes aegypti Red-Eye Genetic Sexing Strains Into Different Genomic Backgrounds for Sterile Insect Technique Applications
Source: Front Bioeng Biotechnol. 2022 Feb 2;10:821428. doi: 10.3389/fbioe.2022.821428 (PMC8847382; doi:10.3389/fbioe.2022.821428)
Supplement: Supplementary file 7 [file Table6.DOCX]

Supplementary Material

# Supplementary Material 6

###Analysis among populations

1. Analysis of Deviance Table

Model: quasibinomial, link: logit

Response: cbind(recombinants, progeny - recombinants)

Terms added sequentially (first to last)

Df Deviance Resid. Df Resid. Dev F Pr(>F)

NULL 73 1286.90

inter 11 1159.3 62 127.55 51.375 < 2.2e-16 ***

---

Signif. codes: 0 ‘***’ 0.001 ‘**’ 0.01 ‘*’ 0.05 ‘.’ 0.1 ‘ ’ 1

2. Pairwise ccomparisons

contrast estimate SE df z.ratio p.value

BRA.RGSS - IDN.RGSS -0.42052 0.139 Inf -3.035 0.0024

BRA.RGSS - LKA.RGSS 0.06082 0.158 Inf 0.385 0.7006

BRA.RGSS - MEX.RGSS -0.46391 0.133 Inf -3.495 0.0005

BRA.RGSS - SGP.RGSS -0.27184 0.189 Inf -1.436 0.1509

BRA.RGSS - THA.RGSS -0.05093 0.148 Inf -0.343 0.7313

BRA.RGSS - BRA.RGSS_35 2.31226 0.399 Inf 5.798 <.0001

BRA.RGSS - IDN.RGSS_35 1.56837 0.261 Inf 6.020 <.0001

BRA.RGSS - LKA.RGSS_35 2.38732 0.399 Inf 5.987 <.0001

BRA.RGSS - MEX.RGSS_35 2.39548 0.332 Inf 7.220 <.0001

BRA.RGSS - SGP.RGSS_35 3.56138 1.019 Inf 3.495 0.0005

BRA.RGSS - THA.RGSS_35 2.39129 0.399 Inf 5.997 <.0001

IDN.RGSS - LKA.RGSS 0.48135 0.141 Inf 3.422 0.0006

IDN.RGSS - MEX.RGSS -0.04339 0.111 Inf -0.390 0.6966

IDN.RGSS - SGP.RGSS 0.14868 0.175 Inf 0.850 0.3953

IDN.RGSS - THA.RGSS 0.36960 0.129 Inf 2.855 0.0043

IDN.RGSS - BRA.RGSS_35 2.73278 0.392 Inf 6.969 <.0001

IDN.RGSS - IDN.RGSS_35 1.98889 0.250 Inf 7.947 <.0001

IDN.RGSS - LKA.RGSS_35 2.80784 0.392 Inf 7.160 <.0001

IDN.RGSS - MEX.RGSS_35 2.81600 0.324 Inf 8.697 <.0001

IDN.RGSS - SGP.RGSS_35 3.98190 1.016 Inf 3.917 0.0001

IDN.RGSS - THA.RGSS_35 2.81181 0.392 Inf 7.171 <.0001

LKA.RGSS - MEX.RGSS -0.52474 0.135 Inf -3.889 0.0001

LKA.RGSS - SGP.RGSS -0.33266 0.191 Inf -1.743 0.0813

LKA.RGSS - THA.RGSS -0.11175 0.150 Inf -0.744 0.4570

LKA.RGSS - BRA.RGSS_35 2.25144 0.400 Inf 5.636 <.0001

LKA.RGSS - IDN.RGSS_35 1.50754 0.262 Inf 5.762 <.0001

LKA.RGSS - LKA.RGSS_35 2.32649 0.399 Inf 5.824 <.0001

LKA.RGSS - MEX.RGSS_35 2.33466 0.333 Inf 7.018 <.0001

LKA.RGSS - SGP.RGSS_35 3.50056 1.019 Inf 3.434 0.0006

LKA.RGSS - THA.RGSS_35 2.33046 0.399 Inf 5.834 <.0001

MEX.RGSS - SGP.RGSS 0.19208 0.170 Inf 1.128 0.2594

MEX.RGSS - THA.RGSS 0.41299 0.123 Inf 3.353 0.0008

MEX.RGSS - BRA.RGSS_35 2.77618 0.390 Inf 7.116 <.0001

MEX.RGSS - IDN.RGSS_35 2.03228 0.247 Inf 8.225 <.0001

MEX.RGSS - LKA.RGSS_35 2.85123 0.390 Inf 7.309 <.0001

MEX.RGSS - MEX.RGSS_35 2.85939 0.321 Inf 8.899 <.0001

MEX.RGSS - SGP.RGSS_35 4.02530 1.016 Inf 3.963 0.0001

MEX.RGSS - THA.RGSS_35 2.85520 0.390 Inf 7.319 <.0001

SGP.RGSS - THA.RGSS 0.22091 0.183 Inf 1.209 0.2266

SGP.RGSS - BRA.RGSS_35 2.58410 0.413 Inf 6.260 <.0001

SGP.RGSS - IDN.RGSS_35 1.84020 0.282 Inf 6.536 <.0001

SGP.RGSS - LKA.RGSS_35 2.65915 0.413 Inf 6.442 <.0001

SGP.RGSS - MEX.RGSS_35 2.66732 0.349 Inf 7.653 <.0001

SGP.RGSS - SGP.RGSS_35 3.83322 1.025 Inf 3.741 0.0002

SGP.RGSS - THA.RGSS_35 2.66312 0.413 Inf 6.452 <.0001

THA.RGSS - BRA.RGSS_35 2.36319 0.396 Inf 5.972 <.0001

THA.RGSS - IDN.RGSS_35 1.61929 0.256 Inf 6.330 <.0001

THA.RGSS - LKA.RGSS_35 2.43824 0.396 Inf 6.162 <.0001

THA.RGSS - MEX.RGSS_35 2.44640 0.328 Inf 7.457 <.0001

THA.RGSS - SGP.RGSS_35 3.61231 1.018 Inf 3.549 0.0004

THA.RGSS - THA.RGSS_35 2.44221 0.396 Inf 6.172 <.0001

BRA.RGSS_35 - IDN.RGSS_35 -0.74390 0.450 Inf -1.653 0.0983

BRA.RGSS_35 - LKA.RGSS_35 0.07505 0.542 Inf 0.139 0.8898

BRA.RGSS_35 - MEX.RGSS_35 0.08321 0.495 Inf 0.168 0.8664

BRA.RGSS_35 - SGP.RGSS_35 1.24912 1.083 Inf 1.153 0.2488

BRA.RGSS_35 - THA.RGSS_35 0.07902 0.542 Inf 0.146 0.8840

IDN.RGSS_35 - LKA.RGSS_35 0.81895 0.450 Inf 1.820 0.0687

IDN.RGSS_35 - MEX.RGSS_35 0.82711 0.392 Inf 2.111 0.0348

IDN.RGSS_35 - SGP.RGSS_35 1.99301 1.040 Inf 1.916 0.0554

IDN.RGSS_35 - THA.RGSS_35 0.82292 0.450 Inf 1.829 0.0674

LKA.RGSS_35 - MEX.RGSS_35 0.00816 0.495 Inf 0.017 0.9868

LKA.RGSS_35 - SGP.RGSS_35 1.17406 1.083 Inf 1.084 0.2784

LKA.RGSS_35 - THA.RGSS_35 0.00397 0.542 Inf 0.007 0.9942

MEX.RGSS_35 - SGP.RGSS_35 1.16590 1.060 Inf 1.100 0.2715

MEX.RGSS_35 - THA.RGSS_35 -0.00419 0.495 Inf -0.008 0.9932

SGP.RGSS_35 - THA.RGSS_35 -1.17010 1.083 Inf -1.080 0.2800
